# Efficacy of swish and gargle and other collection methods with the use of Abbott ID NOW in COVID-19 detection

**DOI:** 10.1017/ash.2025.10192

**Published:** 2025-10-27

**Authors:** Amin Motamed Ektesabi, Greg J. German, Le Luu, Claudia dos Santos, Larissa M. Matukas

**Affiliations:** 1 Institute of Medical Sciences, Faculty of Medicine, University of Toronto, Toronto, ON, Canada; 2 Keenan Research Centre for Biomedical Science, St. Michael’s Hospital, Toronto, ON, Canada; 3 Unity Health Toronto, Toronto, ON, Canada; 4 Department of Laboratory Medicine and Pathobiology, University of Toronto, Toronto, ON, Canada

## Abstract

**Background:**

To evaluate the efficacy of the Abbott ID NOW system in detecting COVID-19 using different specimen collection methods, emphasizing diagnostic accuracy and patient comfort.

**Methods:**

Three cohorts were analyzed, including two using the nasopharyngeal (NP) swab technique and one utilizing the swish-and-gargle (SG) method. Positive percent agreement (PPA), negative percent agreement (NPA), and cycle threshold (Ct) values were assessed to determine the system’s performance.

**Results:**

The PPA for the NP swab cohorts averaged around 70%, while the SG cohort exhibited a higher PPA of 80%. All cohorts maintained high NPAs, close to 100%. The SG method significantly reduced false negatives, especially at lower Ct values, indicative of elevated levels of viral RNA. Additionally, the NP swab method, often uncomfortable, posed challenges in repeated testing scenarios, particularly among healthcare workers.

**Conclusion:**

While the Abbott ID NOW system demonstrates reliable COVID-19 detection, the SG method emerges as a superior collection technique to NP swabs, offering enhanced diagnostic accuracy and improved comfort for test takers. This study underscores the importance of selecting appropriate collection methods to ensure accurate and efficient COVID-19 testing.

## Introduction

In response to the global surge of ever-changing variants of COVID-19, healthcare systems worldwide have been under tremendous pressure to test staff and patients efficiently and routinely.^1^ Although the acute pandemic phase has subsided, COVID-19 remains a persistent public health concern. Ongoing circulation of SARS-CoV-2, including emerging variants, continues to necessitate accessible and reliable testing strategies. As population testing needs shift—from mass testing to more targeted screening in clinical, occupational, and community settings—there is increasing emphasis on collection methods that balance accuracy, speed, and patient comfort. The pandemic experience has also driven clinical laboratories and public health agencies toward rapid, point-of-care (POC) diagnostics with short turnaround times, a shift now embedded in patient and provider expectations. This scenario underscores the need for diagnostic tools with rapid turnaround times. The Abbott ID NOW is a compact POC testing device with ease of use and accessibility in operation. Notably, it delivers positive results in as little as five minutes and negative results within thirteen minutes.^2,3^

Since the onset of the COVID-19 pandemic, numerous studies have investigated the reliability of the Abbott ID NOW in producing accurate COVID-19 test results.^4–6^ Its implementation has been widespread, extending from community hospitals and clinics to large, multisite academic medical centers. This extensive adoption has facilitated timely and precise diagnostic results, promoting equitable access to care and enhancing healthcare delivery across health network systems.^7^ However, despite the rapid results produced by these tests—a feature especially valuable in urgent care settings—several challenges have been identified. These include a lower throughput capacity and the logistical demands associated with the rapid transportation of samples in large healthcare centers.^7^ In emergency departments, these tests have proven instrumental in enabling swift clinical decision-making and the implementation of effective infection control measures.^7^ A notable application of the Abbott ID NOW device is its potential to serve a crucial role in screening healthcare staff before the commencement of their shifts.^8^ Nonetheless, it is important to highlight that the sensitivity of the Abbott ID NOW, particularly when utilizing nasopharyngeal (NP) swab samples, is generally higher when viral loads are elevated. Under conditions of low viral load, the risk of generating false negative results increases. Moreover, healthcare workers (HCWs) have reported discomfort associated with using NP swabs, which may render this device less appealing for routine, daily use.^9^ This subjective discomfort, in combination with the potential for decreased sensitivity under certain conditions, suggests the need for ongoing evaluation and refinement of this testing approach.^10^

This study evaluates the efficacy of various specimen collection methods and detection techniques for COVID-19 with a focus on saline swish and gargle. Swish-and-gargle (SG) can be a more comfortable alternative to more invasive specimen collection methods, but to ensure reproducibility, explicit details on the fluid used, the duration of swishing, and the instructions given to participants are essential. The primary objective is to assess the efficacy of the ID NOW assay test POC test compared to standard laboratory-based tests using alternative sample collection methods. Multiple studies have shown saliva or oral rinse specimens can perform comparably, or in some cases better, than traditional NP swabs in detecting SARS-CoV-2, with differences in viral dynamics over the course of infection.^11^ These findings align with our focus on evaluating saline SG as an alternative collection method.

## Materials and methods

### Inclusion criteria

The inclusion criteria for this quality study encompassed a diverse range of participants, including patients with and without symptoms who were assessed in the emergency department, outpatient assessment clinics, and HCWs associated with the Unity Health Toronto network. Symptomatic individuals of all age groups, within seven days of symptom onset, were included, as were asymptomatic contacts of all ages with a confirmed case of COVID-19 and asymptomatic screening of HCWs. The inclusion of these asymptomatic persons was as directed or recommended by infection prevention and control authorities, public health officials, or institutional occupational health.

### Abbott ID NOW validation

The instrument’s performance was validated in the ISO 15 189-accredited microbiology laboratory at Unity Health Toronto. During patient and specimen assessment, protective measures were followed, including appropriate use of personal protective equipment (PPE) and ensuring that samples were maintained at room temperature, in accordance with the manufacturer’s guidelines, to minimize the risk of false positives. Nasopharyngeal (NP) swabs were collected by trained healthcare professionals and immediately processed using the Abbott ID NOW system, following the manufacturer’s instructions, which included direct swab insertion into the sample receiver cartridge without the use of transport medium. For the SG collection method, participants were instructed to swish and gargle 10 mL of sterile normal saline for 30 s, after which the sample was collected in sterile containers. Trained staff then aliquoted the specimen for immediate testing on the ID NOW platform. Residual SG specimens were aseptically stored in labeled Universal Transport Medium (UTM) tubes and frozen at −80°C for potential future analysis. To validate Abbott ID NOW results, paired samples were tested in parallel using the Seegene Allplex 2019-nCoV assay, which targets the E-gene, RdRp, and N-gene of SARS-CoV-2. While the exact primer/probe sequences are proprietary, these gene targets confirm the presence or absence of the viral genome, though they do not distinguish between variants.

### Statistical analysis

The Abbott ID NOW system (Abbott Laboratories, Chicago, IL) was assessed for accuracy against one of four laboratory-validated polymerase chain reaction (PCR) systems, as previously described.^9^ Positive Percent Agreement (PPA) quantifies how often the device correctly identifies positive results, while Negative Percent Agreement (NPA) measures its accuracy in detecting negatives. Both metrics offer insights into the system’s alignment with PCR findings. To evaluate discrepancies in false negative results, a non-parametric t test (Mann-Whitney test) was performed comparing the NP swab cohort (combined data from cohorts 1 and 2) with the SG cohort (cohort 3). This study was designed as a pragmatic quality improvement initiative using all available specimens during the collection periods; therefore, no formal a priori power calculation was performed. The sample sizes (467, 253, and 1,704 across cohorts) exceed those in several prior evaluations and provide reasonable ability to detect differences, though larger multicenter studies would be needed for confirmation.

## Results

The initial cohort of 467 specimens was collected from voluntary outpatient participants, including both symptomatic individuals and asymptomatic contacts, who attended the St. Michael’s Hospital (SMH) and St. Joseph’s Health Centre (SJHC) COVID-19 assessment centers. The collection period for this cohort spanned from January 15th, 2021, to February 26th, 2021. For each participant, two NP swabs were collected simultaneously—one designated for testing on the Abbott ID NOW system and the other submitted for laboratory-based PCR testing, depending on availability. The overall community disease prevalence for this cohort was 6.4%. Out of the asymptomatic population that underwent testing (*n* = 194), the ID NOW system accurately identified 8 out of 11 true positives, resulting in a PPA of 73%. All individuals who were true negatives were correctly diagnosed as negative, yielding an NPA of 100%. When considering the entire cohort (30 PCR-confirmed positives), the PPA was 76.7% and the NPA remained 100%, as shown in Table 1. A comprehensive breakdown of the PPA and NPA for the Abbott ID NOW system within the first cohort is presented in Table 1.

**Table 1.** Diagnostic performance: PPA and NPA metrics

Cohort 1	NP swab (Jan 15^th^ to Feb 26^th^, 2021)		
Total specimen collected (outpatients + volunteered HCW)			467
	True positive (PCR)		30
	ID NOW true positives		23
	ID NOW false positives		0
	ID NOW false negatives		7
	Cohort PPA		76.67%
	NPA		100%
Cohort 2	**NP swab (Jan 17** ^**th**^ **to Feb 4** ^**th**^ **, 2022)**		
Total specimen collected (HCWs)			253
	True positive (PCR)		25
	ID NOW true positives		17
	ID NOW false positives		2
	ID NOW false negatives		8
	Cohort PPA		68.00%
	NPA		99%
Cohort 3	**Swish and gargle (Feb 9** ^**th**^ **to June 17** ^**th**^ **, 2022)**		
Total specimen collected (HCWs)			1704
	True positive (PCR)		110
	ID NOW true positives		88
	ID NOW false positives		0
	ID NOW false negatives		22
	Cohort PPA		80%
	NPA		100%

The second cohort, composed exclusively of symptomatic and asymptomatic HCWs, included 253 paired NP swabs collected between January 17th, 2022, and February 4th, 2022. For each participant, two NP swabs were collected—one processed using the Abbott ID NOW system and the other submitted for laboratory-based PCR testing. The overall community disease prevalence for this cohort was 9.7%. Of the 25 PCR-confirmed positive cases, the ID NOW system correctly identified 17 and missed 8, resulting in a PPA of 68%. Among the 231 PCR-confirmed negatives, 229 were accurately identified and 2 were false positives, yielding an NPA of 99%. A detailed breakdown of the PPA and NPA for the Abbott ID NOW system in this second cohort is presented in Table 1. The PPA and NPA values for cohorts 1 and 2 are remarkably consistent. Thus, despite being collected at different time points, we merged these two groups for subsequent analyses and refer to them collectively as the NP swab cohort. The third and final cohort consisted of 1,704 samples collected between February 9th, 2022, and June 17th, 2022. In this cohort, we collected samples from asymptomatic HCWs who intended to work at the SMH facility prior to the start of their shift. For this cohort, the SG method was employed for specimen collection for the Abbott ID NOW system, diverging from the traditional NP swab technique. Two separate SG samples were collected from each participant—one tested using the Abbott ID NOW system and the other submitted for laboratory-based PCR analysis. The community disease prevalence for this cohort was ascertained to be 6.4% at the time of assessing ID NOW. Out of the 110 true positives, the ID NOW system accurately diagnosed 88, resulting in a PPA of 80%, significantly higher than the NP swab cohort at 68%. No false positives were reported in this cohort. Therefore, the NPA remained at 100%. Across cohorts, PPV/NPV were: Cohort 1, 100%/98.4%; Cohort 2, 89.5%/96.6%; Cohort 3, 100%/98.6%.

### Performance breakdown of Abbott ID NOW

**Figure 1.**
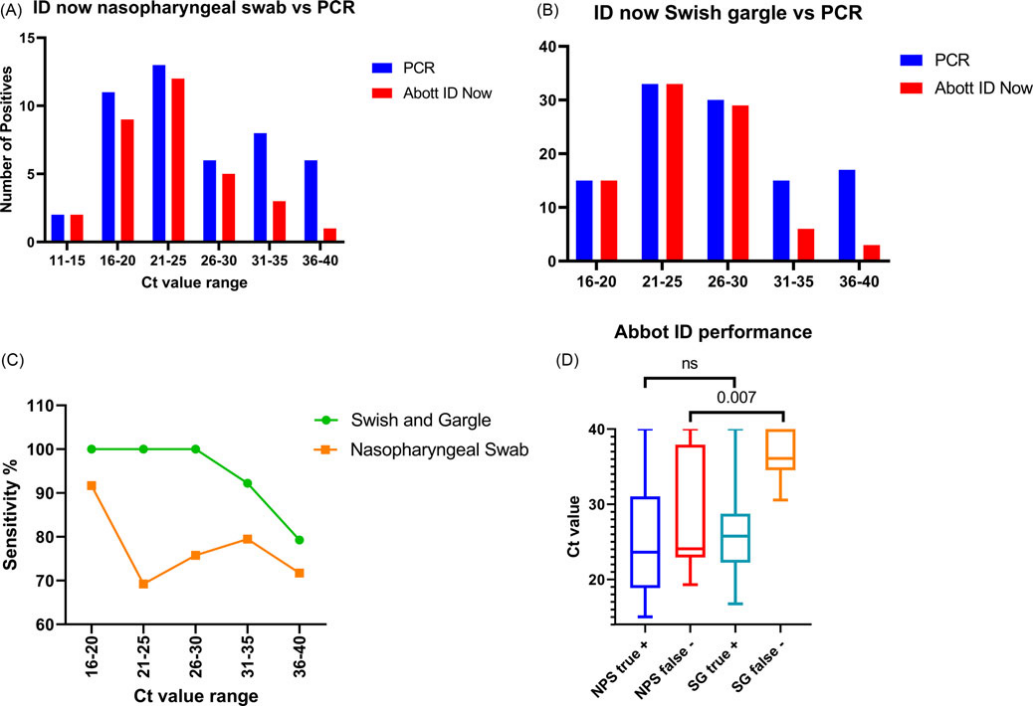
Performance breakdown of Abbott ID NOW. **A**: False negatives in the NP swab cohort across Ct value intervals. **B**: False negatives in the SG swab cohort across Ct value intervals. **C**: Sensitivity comparison between NP and SG samples across Ct value intervals. **D**: Graph comparing false negative Ct values between NP swab and SG collection methods.

To assess the reliability of results based on the cycle threshold (Ct), cycle count needed for the fluorescent signal to surpass a set threshold, and to pinpoint potential shortcomings of the device, we segmented the number of true positives produced by the Abbott ID NOW and PCR into intervals of 5 cycle thresholds. This analysis is crucial since the Ct is associated with the number of viral genomic copies harnessed by the ID NOW to deliver a positive diagnosis. In the NP swab cohort, false negatives were observed with Ct values ranging from 16 to 40 (Figure 1A). Contrasting this with data from the SG cohort, it is evident that false negatives arise at considerably higher cycle thresholds (and weaker positive reactions), specifically between 31 and 40 (Figure 1B). These findings suggest that the sample collection method can directly influence the device’s ability to correctly diagnose a positive specimen, especially when viral copies are present in higher numbers. This also elucidates why the PPA is higher in the SG cohort compared to the NP swab cohort (80% vs 68–77%, with an overall average of ∼ 72%).

Diving into the sensitivity variations between the two cohorts shows that the Abbott ID NOW exhibits reduced sensitivity with the NP collection method. Samples from the NP cohort consistently showed ∼ 70–80% sensitivity across lower Ct intervals, but the sensitivity began to decline after Ct values surpassed ∼ 21–25. In contrast, sensitivity remained stable (100%) in the SG cohort and only decreased after the Ct value of 30. Across all Ct value ranges, the sensitivity of the Abbott ID NOW device was consistently higher when samples were procured using the SG method (Figure 1C).

To examine the discrepancies in false negative Ct values between NP samples vs SG samples. We analyzed the qPCR Ct values associated with each false negative identified in both groups. A non-parametric t test was used to determine if the SG collection methodology significantly reduces the probability of encountering false negatives, particularly in samples with lower Ct values. The SG method significantly reduced the likelihood of recording false negatives at lower Ct values, with a *P* value of .007 compared to the NP cohort. It’s also worth noting that the difference in true positive detection between the two methods remained statistically insignificant. This suggests that while the SG method enhances the system’s proficiency in minimizing false negatives, it does not compromise the Abbott ID NOW device’s capacity to detect true positive specimens accurately (Figure 1D).

## Discussion

The primary objective of this study was to evaluate the efficacy of the Abbott ID NOW system in detecting COVID-19 across patient and HCW populations and collection methods. Our findings offer significant insights into this testing device’s strengths and potential shortcomings, providing valuable information for healthcare and diagnostic institutions. The PPA and NPA values observed in the first two cohorts (nasopharyngeal swabs) were consistent despite the minor discrepancies in community disease prevalence and mix of symptomatic and asymptomatic participants. The decision to merge these cohorts for subsequent analyses seemed reasonable, given their methodological similarities in sample collection. Nonetheless, because these NP swab data were collected at different time points and under different prevalence conditions, the positive detection rates may be influenced by factors such as viral load distribution, patient disease status, and circulating variants. This limitation warrants caution in generalizing the direct comparison of NP results to the SG results. Moving on to the SG method, it was observed that the Abbott ID NOW system’s performance significantly improved when using the SG method over the NP swab technique. The elevated PPA of 80% in the SG cohort, compared to around 70% in the NP swab cohort, underscores the influence of collection methods on diagnostic accuracy. Our findings are consistent with recent literature demonstrating high sensitivity for saliva-based sampling, including a study showing that saliva offers both high diagnostic accuracy and distinct viral kinetics compared to nasal swabs.^11^ This supports the role of non-invasive oral sampling in meeting the evolving demands for rapid, patient-friendly testing in clinical and occupational settings.

Another aspect that must be taken into consideration is the patient’s experience during specimen collection. NP swab collection, often uncomfortable, can be particularly distressing and painful for some individuals. This discomfort becomes even more pronounced for HCWs who undergo frequent testing. If there is a recurring need for COVID-19 testing among healthcare professionals, it is essential to adopt a method that is accurate and well-tolerated by those tested. The SG method appears to offer a more patient-friendly alternative in this regard and is supported by other studies.^9–11^

Another important consideration is the interpretation of Ct values in PCR testing. While Ct values correlate with the amount of viral genomic material present in a specimen, they are not absolute quantitative measures, and PCR remains a qualitative diagnostic tool in this context. Ct values are influenced by assay platform, specimen type, and handling, and should therefore be regarded as semi-quantitative indicators rather than precise measures of viral load. Unlike PCR, the Abbott ID NOW system uses isothermal amplification and does not produce Ct values. Our data revealed that the NP swab cohort had false negatives spanning a wide range of Ct values, from 16 to 40, whereas the SG cohort primarily experienced false negatives at higher Ct values, from 31 to 40. This suggests that the SG method may be more effective in detecting low viral loads that still fall within the detectable range of ID NOW. Moreover, the reduced sensitivity observed with NP swabs, particularly at Ct values above 21, reinforces the impact of specimen collection techniques on diagnostic performance. In contrast, the SG method demonstrated greater consistency and sensitivity even at higher Ct values, supporting its potential as a more reliable collection method for use with POC diagnostics.

Our statistical analysis comparing false negative Ct values between the NP swab and SG cohorts provided more insight into the efficacy of these collection methods. The SG method’s significant reduction in the probability of encountering false negatives from 68% in cohort 2 to 80% in cohort 3, particularly in samples with lower Ct values lower than 30, supports its superior performance over the NP swab method.^10^ Despite this, it’s important to acknowledge that the Abbott ID NOW system maintains its accuracy in identifying true positives across various collection methods. This consistency is crucial for the timely identification of infected individuals and the implementation of appropriate interventions.

It is also important to note that the number of truly positive samples is limited in each Ct interval, especially for the NP swab cohorts, making sub-analyses prone to wide confidence intervals. This is a key limitation of our work and underscores that larger studies are needed to confirm the observed performance differences. Additionally, future investigations should control for the time postsymptom onset, as well as patient demographics and variants in circulation, to ensure more robust comparisons.

In conclusion, our research reinforces the significant impact that collection methods have on diagnostic accuracy. While the Abbott ID NOW system remains effective for COVID-19 detection, the SG technique emerges as a more effective collection strategy for HCWs or any individuals who need to be tested frequently. This approach not only minimizes false negatives but also enhances patient comfort. Healthcare institutions should consider these findings when selecting the most appropriate collection method, aiming to improve accuracy in COVID-19 detection and ensuring the well-being of individuals undergoing testing. Future studies are encouraged to explore additional factors that may affect the performance of testing systems, helping to maintain a proactive stance in the ongoing battle against the pandemic.
